# Supplementary material for: Content-rich biological network constructed by mining PubMed abstracts
Source: BMC Bioinformatics. 2004 Oct 8;5:147. doi: 10.1186/1471-2105-5-147 (PMC528731; doi:10.1186/1471-2105-5-147)
Supplement: Additional File 2 — The original results of the above study (non-essential files are deleted to keep the file size under the limit set by BMC bioinformatics). [file 1471-2105-5-147-S2.bz2 › chilibotAdditionalFile2/dip10/136ID8872303E489/html/GAP43_CALM1.html]

 


 **GAP43** and **CALM1** 
  
Found 143 abstracts in PubMed, retrieved 10.  
 

 What does Google say? 
 PDF only 
| .edu only 

---

**Interactive relationship** (e.g. stimulation, inhibition, etc)

**Inhibitory relationship**- Administration of palmitoylcarnitine, although did not change the subcellular distribution of GAP 43  [ **GAP43** ] , decreased its phosphorylation, which could regulate other signal transduction pathways calmodulin  [ **CALM1** ]  and G 0 dependent.  Ref: 12061781 Biochem Biophys Res Commun, 2002
**Neutral relationship**- Apo calmodulin  [ **CALM1** ]  binds typical target sequences from skeletal muscle myosin light chain kinase and neuromodulin  [ **GAP43** ]  with Kd approximately 70 90 nM at low ionic strength.  Ref: 11206069 Protein Sci, 2000
- Neuromodulin  [ **GAP43** ]  GAP 43  [ **GAP43** ] , neurogranin RC3, and PEP 19 are small acid stable proteins that bind calcium poor calmodulin  [ **CALM1** ]  through a loosely conserved IQ motif.  Ref: 11414283 Mol Neurobiol< MedlineTA>Mol Neurobiol, 2000

**Non-interactive relationship** (e.g. studied together, co-existance, homology, etc.)

- Because brain derived raft contains two calmodulin  [ **CALM1** ]  binding proteins, GAP 43  [ **GAP43** ]  and NAP 22 as the major protein components, the raft domain is assumed to be important in the Ca 2 signaling.  Ref: 11319767 J Neurosci Res, 2001
- The proteolysis disturbs some properties characteristic for whole GAP 43  [ **GAP43** ]  molecules, in particular, calmodulin  [ **CALM1** ]  binding and Ser 41 phosphorylation, when the cleavage occurs at 41st residue.  Ref: 11274743 Neurosci Res, 2001
- Although neuromodulin  [ **GAP43** ] , neurogranin, and PEP 19 were early representatives of the small IQ motif containing protein family, newer examples have come to light that expand the number of cellular systems through which the IQ peptide calmodulin  [ **CALM1** ]  interaction could regulate biological processes including gene transcription.  Ref: 11414283 Mol Neurobiol< MedlineTA>Mol Neurobiol, 2001
- Small proteins that modulate calmodulin  [ **CALM1** ]  dependent signal transduction effects of PEP 19, neuromodulin  [ **GAP43** ] , and neurogranin on enzyme activation and cellular homeostasis.  Ref: 11414283 Mol Neurobiol< MedlineTA>Mol Neurobiol, 2001
- To better understand the molecular basis of this neuroanatomical alteration, the present study examined three developmentally regulated protein kinase C substrate mRNAs that are highly expressed in hippocampal granule cells during mossy fiber outgrowth GAP 43  [ **GAP43** ] , a presynaptic substrate implicated in axonal outgrowth, RC3 neurogranin, a postsynaptic substrate implicated in calmodulin  [ **CALM1** ]  signaling, and MARCKS like protein MLP, which binds calmodulin  [ **CALM1** ]  and filamentous actin in neurons and glial cells.  Ref: 12697986 Dev Neurosci, 2002
